# Updates in Contemporary Management of Singleton Pregnancies Complicated by a Short Cervix

**DOI:** 10.3390/jcm14155544

**Published:** 2025-08-06

**Authors:** Moti Gulersen, Vincenzo Berghella, Eran Bornstein

**Affiliations:** 1 Division of Maternal-Fetal Medicine, Department of Obstetrics and Gynecology, Sidney Kimmel Medical College, Thomas Jefferson University, Philadelphia, PA 19107, USA; 2 Division of Maternal-Fetal Medicine, Department of Obstetrics and Gynecology, Lenox Hill Hospital—Zucker School of Medicine at Hofstra/Northwell, New York, NY 11549, USA

**Keywords:** preterm birth, prematurity, cervical length, neonatal morbidity, cerclage, vaginal progesterone, cervical pessary

## Abstract

Singleton pregnancies complicated by a short cervical length (≤25 mm) are at significantly increased risk for spontaneous preterm birth. Several treatment strategies aimed at reducing this risk and improving perinatal outcomes have been evaluated, including vaginal progesterone, cervical cerclage, and cervical pessary. This review summarizes the latest evidence regarding the efficacy of these interventions. Vaginal progesterone and/or cervical cerclage have been identified as proven evidence-based practices for preterm birth prevention and improve neonatal outcomes. Vaginal progesterone reduces the risk of preterm birth < 35 weeks by 27% (relative risk 0.73, 95% confidence interval 0.58–0.90). Cervical cerclage has been shown to reduce the risk of preterm birth < 35 weeks by 30% (relative risk 0.70, 95% confidence interval 0.55–0.89) in patients with a short cervical length and prior preterm birth. In contrast, recent data suggest that cervical pessary should no longer be considered a management option for these patients. A continued focus on individualized, evidence-based approaches remains essential to optimizing outcomes in this high-risk population.

## 1. Introduction

Preterm birth remains a leading cause of neonatal morbidity and mortality worldwide, affecting approximately 10% of pregnancies [1]. Its global burden includes immediate perinatal complications, long-term health consequences for affected children, and a substantial economic burden on healthcare systems [1]. Transvaginal ultrasound screening of the cervical length (CL) has long been considered a useful tool for identifying patients at risk for spontaneous preterm birth [2,3]. All pregnant individuals should undergo a CL evaluation via transvaginal ultrasound [4], with the timing of initial assessment guided by the presence or absence of risk factors for spontaneous preterm birth (i.e., a history of prior spontaneous preterm birth). Given the limited accuracy and reproducibility of transabdominal CL measurements, transvaginal ultrasound is the preferred and recommended modality [3,5,6]. In low-risk patients, a CL measurement should be performed at the detailed fetal anatomical evaluation, typically between 18 0/7 and 21 6/7 weeks of gestation. For patients at high risk of spontaneous preterm birth, individualizing serial CL surveillance should be considered, beginning as early as 14–16 weeks [3,7,8].

A short cervix, most commonly defined as CL ≤ 25 mm during the midtrimester, is associated with a significantly increased risk of spontaneous preterm birth [2,9,10]. Moreover, the relationship between CL and incidence of preterm birth is inverse; the shorter the CL, the higher the risk of preterm birth [11]. Several treatment options aimed at preventing preterm birth have been explored with varying degrees of success in asymptomatic individuals identified at risk [3]. Moreover, management strategies may vary depending on the presence of other risk factors for preterm birth, such as a prior spontaneous preterm birth [3]. These include vaginal progesterone, cervical cerclage, and cervical pessary. Use of cervical elastography and serum evaluations of inflammatory biomarkers have been proposed as screening strategies to assess risk of preterm birth regardless of CL measurement and are outside the scope of this review [12,13]. Additionally, use of antibiotics for treatment of short cervix has been investigated in few observational studies, and evidence demonstrating benefit associated with its routine use is lacking [14]. While a short cervix may be a common pathway for several etiologies leading to preterm birth [15], none of the acceptable treatment options completely eliminate the risk of preterm birth and different patients may benefit from different interventions. Despite widespread use of CL screening and preterm birth preventative interventions, gaps remain in clinical practice regarding optimal patient selection, timing, and choice of therapy, particularly for those without a prior preterm birth. The purpose of this review is to summarize the latest evidence on management strategies to reduce the risk of preterm birth in singleton pregnancies complicated by a short cervix.

## 2. Vaginal Progesterone

The use of vaginal progesterone for the prevention of preterm birth in patients with a short cervix has been evaluated in several randomized clinical trials and validated in multiple studies with patients from different geographical regions as well as ethnic and racial groups [16,17]. The rationale for its initial use was derived from its essential role as a hormone in maintaining pregnancy and promoting uterine and cervical quiescence [18,19]. A decline in progesterone is thought to contribute to cervical shortening and subsequently preterm labor [19,20]. Thus, administration of vaginal progesterone was hypothesized to prevent this progression. In a systematic review and meta-analysis including individual patient data (IPD) from 5 randomized trials, Romero et al. reported that vaginal progesterone reduced the risk of preterm birth prior to 33 weeks in singletons with a short CL ≤ 25 mm (14% vs. 22%; relative risk [RR] 0.62, 95% confidence interval [CI] 0.47–0.81) [16]. Vaginal progesterone also improved neonatal outcomes by reducing the risk of respiratory distress syndrome (5% vs. 10%; RR 0.47, 95% CI 0.27–0.81) and composite neonatal morbidity and mortality (8% vs. 14%; RR 0.59, 95% CI 0.38–0.91) (Table 1). Approximately 75% of patients in the analysis had no prior preterm birth and a CL between 10 and 20 mm. Although the daily dose of vaginal progesterone included in the trials ranged from 90 to 200 mg and formulation varied (i.e., gel or micronized capsule), most patients received 200 mg of micronized progesterone capsules. Treatment was typically initiated between 18 and 25 weeks of gestation and continued until 34–36 weeks. Of note, this meta-analysis included data from the OPPTIMUM study [21], a clinical trial from the United Kingdom that raised questions about the clinical efficacy of progesterone for preterm birth prevention after demonstrating no benefit with its use compared to placebo in patients with a short CL ≤ 25 mm. The authors recently repeated an IPD meta-analysis with exclusion of the trial by Cetingoz et al. after its retraction due to concerns regarding their method of randomization and similar findings were observed [22].

In addition to the evidence of preterm birth reduction in patients treated by vaginal progesterone, long-term follow-up data have been reassuring with no major safety concerns. The OPPTIMUM study reported no significant differences in neurodevelopmental outcomes at 2 years of age between children exposed in utero to progesterone compared to placebo [21]. Similar findings of no increased risk of neurodevelopmental impairment were reported by O’Brien et al., who evaluated such outcomes at 6, 12, and 24 months of age following exposure [23].

Although several factors contribute to the preterm labor syndrome, evidence from systematic reviews and meta-analyses has consistently demonstrated benefit of vaginal progesterone for preterm birth prevention. Thus, this treatment strategy is considered standard of care and recommended by the Society of Maternal-Fetal Medicine (SMFM), American College of Obstetricians and Gynecologists (ACOG), Society of Obstetricans and Gynaecologists of Canada (SOGC), International Federation of Gynecology and Obstetrics (FIGO), National Institute for Health and Care Excellence (NICE), and World Health Organization (WHO). Therefore, we, and many others offer this treatment as the first-line intervention for asymptomatic low-risk patients with an incidental finding of CL ≤ 25 mm [3,5,24,25].

## 3. Cervical Cerclage

It has been nearly 70 years since commonly used cervical cerclage techniques were first described by Drs. Vithal Shirodkar and Ian McDonald [26,27]. The rationale for its initial use was based on findings suggesting that structural weakness or insufficiency of the cervix can lead to cervical shortening, painless cervical dilation, and subsequent preterm birth [28]. Thus, placement of a cerclage in patients with a short cervix was hypothesized to provide mechanical support and prolong pregnancy [29]. Since cervical cerclage has been studied separately in patients with and without a history of prior preterm birth, this review will present the latest evidence in two parts.

## 4. Short Cervix and Prior Preterm Birth

The utility of cerclage for the prevention of preterm birth in patients with a short cervix and prior spontaneous preterm birth has been studied in several well designed randomized trials and is well established [30]. An IPD meta-analysis from 5 randomized trials demonstrated a significant reduction in preterm birth < 35 weeks for patients with a short CL < 25 mm and prior spontaneous preterm birth screened between 14 and 27 weeks who underwent cerclage compared to no cerclage (28.4% vs. 41.3%; RR 0.70, 95% CI 0.55–0.89) (Table 2) [30]. Cerclage also reduced the risk of early preterm birth and composite perinatal mortality and morbidity (15.6% vs. 24.8%; RR 0.64, 95% 0.45–0.91) (Table 2). Although ACOG currently recommends cerclage placement for patients with a short cervix and prior preterm birth < 34 weeks of gestation [31], the upper gestational age limit of prior preterm birth included in the meta-analysis of Berghella et al. was 36 weeks [30]. Therefore, it is our recommendation that providers consider these patients as candidates for cerclage as well.

## 5. Short Cervix and No Prior Spontaneous Preterm Birth

The utility of cerclage for the prevention of preterm birth in patients with an incidental finding of short cervix and no prior spontaneous preterm birth is less clear. Data from an IPD meta-analysis of 5 randomized trials showed that cerclage did not reduce the risk of preterm birth or improve neonatal outcomes in this patient population (Table 2) [32]. However, planned subgroup analyses revealed a significant decrease in preterm birth < 35 weeks in patients who had a cerclage with CL < 10 mm (n = 126 [76 with cerclage and 50 without cerclage], 39.5% vs. 58%; RR 0.68, 95% CI 0.47–0.98) and when tocolytics and antibiotics were used as additional therapy. Observational studies have since also suggested that cerclage may be effective in patients with a CL ≤ 10 mm or progressively shortening CL after initiation of vaginal progesterone and no prior preterm birth [33,34]. It is important to note that these studies have included patients with various degrees of cervical dilation. Nevertheless, patients with an extremely short cervix represent a higher risk group, where vaginal progesterone may not be as effective alone in preventing preterm birth. As a result of data showing increased latency and decreased preterm birth, the Society of Maternal-Fetal Medicine (SMFM) have suggested that cerclage placement can be considered for patients with a CL < 10 mm based on shared decision-making [5].

Since report of SMFM’s recommendations, a multicenter international randomized trial focusing exclusively on patients with a short CL and no prior preterm birth was published [35]. Among the 90 patients with a CL ≤ 25 mm between 18 0/7 and 23 6/7 weeks of gestation included in the analysis, cerclage did not reduce the risk of preterm birth < 35 weeks compared to no cerclage (16.3% vs. 23.4%; RR 0.70, 95% CI 0.30–1.63). However, the use of cerclage did result in a significantly longer latency from randomization to delivery (median difference 13 days; *p* = 0.01) and a significantly later gestational age delivery (median difference 1.0 weeks, *p* = 0.035). The trial was stopped early due to lagging enrollment over a 6-year study period. Subgroup analyses were limited by the small sample size to draw conclusions regarding cerclage effectiveness based on CL at the time of randomization. Of note, vaginal progesterone was used in both groups, an intervention that was not utilized in any of the trials included in the initial IPD meta-analysis [32]. More data are needed before universally recommending cerclage for preterm birth prevention in patients with a short CL and no prior spontaneous preterm birth. Nevertheless, it is our practice to recommend vaginal progesterone as first-line treatment in this patient population and perform serial CL surveillance. In cases where the CL is very short (<10 mm), we counsel patients about the potential benefit from cerclage, review the limited data to support it, and utilize shared decision-making. Similar to the SMFM suggestion, we also believe that until additional data from clinical trials are available, it is reasonable to perform cerclage in patients with CL < 10 mm and no prior preterm birth.

While evaluating data related to cerclage technique and adjuvant therapies is outside the scope of this review, we support the use of either McDonald or Shirodkar cerclage with monofilament or braided suture. We acknowledge that choice of technique and suture type may vary based on provider experience, institutional protocols, and regional practice patterns.

## 6. Cervical Pessary

The cervical pessary, commonly referred to as the Arabin pessary, was developed with the thought of reducing preterm birth through mechanisms such as displacing weight of the gravid uterus, altering the uterine–cervical angle, preventing internal cervical os dilation, and protection of the mucus plug [36]. Several randomized trials have evaluated the efficacy of cervical pessary for preterm birth prevention in patients with a short CL with conflicting results [37,38,39]. Nevertheless, a recent systematic review and meta-analysis demonstrated no significant differences in risk of preterm birth or adverse perinatal outcomes in singletons with a short cervix who were randomized to pessary compared to no pessary [40]. The incidences of vaginal discharge and pelvic discomfort, known side effects of pessaries used for the treatment of pelvic organ prolapse, were significantly increased in the pessary group. Differences in timing of pessary placement, frequency of early removal, provider experience, and CL monitoring are proposed reasons for the conflicting results reported in the included trials [40]. For example, provider experience with insertion may impact benefit if incorrectly placed. Early removal due to side effects or patient discomfort may reduce exposure duration, which can also impact efficacy. A recent multicenter clinical trial evaluating pessary in patients with a short cervix confirmed the absence of benefit in preventing preterm birth and has suggested possible harm with its use [41]. Among the 542 patients included in the interim analysis by Hoffman et al., pessary in singletons with a short CL ≤ 20 mm detected between 16 0/7 and 23 6/7 weeks and no prior preterm birth did not reduce the risk of the preterm birth or fetal death < 37 weeks (45.5% vs. 45.6%; RR 1.00, 95% CI 0.83–1.20) [41]. However, the risk of fetal or neonatal/infant death was significantly increased (13.3% vs. 6.8%; RR 1.98, 95% CI 1.13–3.32) in the pessary group compared to control, and thus the trial was halted. The mechanism for this increased risk is unclear but may have been attributed to the fact that patients with fetal or neonatal/infant deaths more commonly had severe CL shortening found early in gestation [41]. Furthermore, the authors proposed several reasons for the differing results from previous trials, including their use of a shorter qualifying CL (<20 mm), earlier gestational age at randomization, and exclusion of patients with a prior preterm birth. Taken together, these data suggest that cervical pessary should no longer be considered a management option for preterm birth prevention in singletons with a short cervix.

## 7. Future Research

Future research should include exploring the expansion of gestational age limits for use of preterm birth prevention strategies. Currently, CL surveillance at 24 weeks or later in asymptomatic patients is not routinely recommended due to insufficient evidence evaluating the utility of interventions for preterm birth prevention at that gestational age. However, a short CL may be identified in patients at later gestational ages [11,42], where the risks of preterm birth and neonatal morbidity and mortality remain significant [43]. This rather arbitrary gestational age threshold, in the past commonly referred to as fetal viability [44], has not been proven based on well-designed studies evaluating the safety and efficacy of such interventions in these settings.

Recent observational data have suggested potential benefit associated with vaginal progesterone use in patients with arrested preterm labor after 24 weeks [45]. Randomized trials evaluating the use of vaginal progesterone exclusively in this patient population are needed. A recent meta-analysis of randomized controlled trials using IPD from 131 patients with a short CL between 24 0/7 and 26 6/7 weeks of gestation reported that cerclage did not result in a statistically significant reduction in preterm birth < 37 weeks (27.3% vs. 38.5%, RR 0.78, 95% CI 0.37–1.28) [46]. However, there was a non-statistically significant decrease of 22% in preterm birth associated with cerclage placement, which is a similar magnitude to the reduction often associated with ultrasound-indicated cerclage before 24 weeks, suggesting that their sample size may have been underpowered to detect this change. A larger randomized controlled trial evaluating the utility of cerclage after 24 weeks is underway.

Combination therapy for cerclage and progesterone has also emerged as a potential meaningful practice for preterm birth prevention. Recent data have suggested use of progesterone with cerclage is associated with reduced rates of preterm birth compared to cerclage alone or progesterone alone [47]. Patients with ultrasound-indicated or physical examination-based cerclage may benefit more from this strategy [47]. However, more data are needed to strengthen this association.

Although use of artificial intelligence has gained popularity in obstetric research [48], its application to the management of short cervix has not been well established. Potential uses include developing a reliable and reproducible automated method to improve accuracy of CL measurement, enhanced risk stratification for preterm birth by integrating clinical and imaging data and identifying patients most likely to benefit from vaginal progesterone or cerclage.

## 8. Conclusions

**Figure 1:**
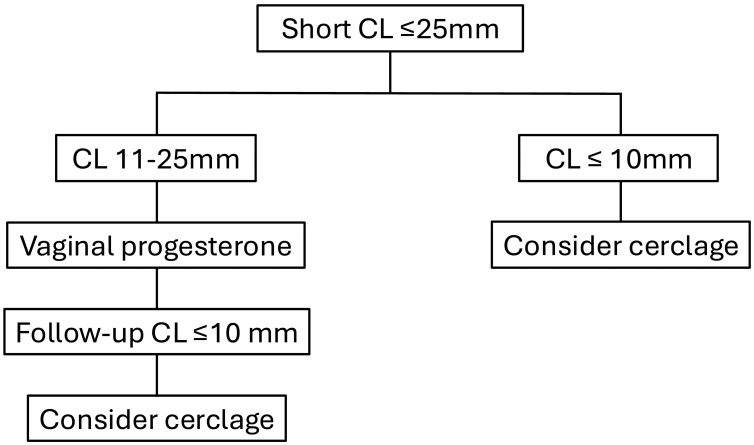
Suggested algorithm for management of patients with a short cervical length and no prior spontaneous preterm birth.

**Figure 2:**
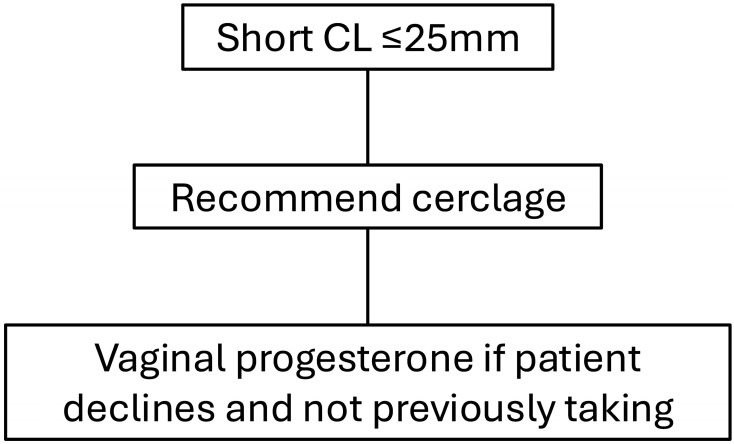
Suggested algorithm for management of patients with a short cervical length and prior spontaneous preterm birth.

Patients with singleton pregnancies and a short cervix present one of the most common high-risk obstetrical dilemmas given the associated risks of preterm birth and adverse perinatal outcomes. Management strategies such as increased surveillance, administration of vaginal progesterone, and/or placement of a cervical cerclage have been identified as proven evidence-based practices for preterm birth prevention and improve neonatal outcomes. Data have suggested that cervical pessary should no longer be considered a management option for these patients. Our recommended management strategies for patients with a short cervix based on their obstetrical history are presented in Figure 1 and Figure 2. A continued focus on individualized, evidence-based approaches remains essential to optimizing outcomes in this high-risk population.

## Figures and Tables

**Table 1:** Preterm birth and adverse perinatal outcomes compared between patients with a short cervix randomized to vaginal progesterone or placebo.

Outcome	Number of Trials Included	Vaginal Progesterone Group	Placebo Group	Relative Risk (95% Confidence Interval)
Preterm birth < 35 weeks	4	105/494 (21%)	138/472 (29%)	0.73 (0.58–0.90)
Preterm birth < 34 weeks	4	85/494 (17%)	125/472 (26%)	0.65 (0.51–0.83)
Preterm birth < 32 weeks	4	62/494 (13%)	91/472 (19%)	0.65 (0.48–0.87)
Respiratory distress syndrome	3	16/361 (4%)	36/354 (10%)	0.45 (0.26–0.80)
Necrotizing enterocolitis	4	11/491 (2%)	12/471 (3%)	0.89 (0.41–1.93)
Intraventricular hemorrhage	4	5/490 (1%)	10/471 (2%)	0.50 (0.18–1.38)
Perinatal death	4	15/494 (3%)	22/472 (5%)	0.64 (0.34–1.22)
NICU admission	4	82/492 (17%)	115/470 (24%)	0.69 (0.53–0.88)
Composite neonatal morbidity and mortality *	3	28/361 (8%)	48/354 (14%)	0.58 (0.37–0.90)

Data are presented as n/n (%). NICU, neonatal intensive care unit. * Includes the occurrence of any of the following events: respiratory distress syndrome, intraventricular hemorrhage, necrotizing enterocolitis, proven neonatal sepsis, or neonatal death.

**Table 2:** Preterm birth and adverse perinatal outcomes compared between patients with a short cervix randomized to cerclage or no cerclage.

Outcome	Number of Trials Included	Cerclage Group	No Cerclage Group	Relative Risk (95% Confidence Interval)
Prior preterm birth				
Preterm birth < 37 weeks	5	105/250 (42.0)	154/254 (60.6)	0.70 (0.58–0.83)
Preterm birth < 35 weeks	5	71/250 (28.4)	105/254 (41.3)	0.70 (0.55–0.89)
Preterm birth < 32 weeks	5	48/250 (19.2)	75/254 (29.5)	0.66 (0.48–0.91)
Preterm birth < 28 weeks	5	32/250 (12.8)	51/254 (20.1)	0.64 (0.43–0.96)
Respiratory distress syndrome	4	13/207 (6.3)	21/196 (10.7)	0.61 (0.32–1.19)
Necrotizing enterocolitis	4	1/207 (0.5)	2/196 (1.0)	0.62 (0.08–4.67)
Intraventricular hemorrhage	4	0/207 (0)	4/196 (2.0)	0.28 (0.05–1.64)
Perinatal mortality	5	22/250 (8.8)	35/254 (13.8)	0.65 (0.40–1.07)
NICU admission	4	57/207 (27.5)	67/196 (34.2)	0.63 (0.34–1.18)
Composite perinatal mortality or morbidity	5	39/250 (15.6)	63/254 (24.8)	0.64 (0.45–0.91)
No prior preterm birth				
Preterm birth < 37 weeks	5	81/224 (36.2)	80/195 (41.0)	0.93 (0.73–1.18)
Preterm birth < 35 weeks	5	49/224 (21.9)	54/195 (27.7)	0.88 (0.63–1.23)
Preterm birth < 32 weeks	5	38/224 (17.0)	39/195 (20.0)	0.96 (0.64–1.42)
Preterm birth < 28 weeks	5	26/224 (11.6)	22/195 (11.3)	1.15 (0.68–1.93)
Respiratory distress syndrome	2	2/14 (14.3)	2/16 (12.5)	1.33 (0.23–7.74)
Necrotizing enterocolitis	2	0/14 (0)	0/16 (0)	–––
Intraventricular hemorrhage	2	1/14 (7.1)	0/16 (0)	3.90 (0.18–85.93)
Neonatal death	4	7/118 (5.9)	6/92 (6.5)	1.08 (0.41–2.86)
NICU admission	3	3/67 (4.5)	4/38 (10.5)	0.80 (0.26–2.47)

Data are presented as n/n (%). NICU, neonatal intensive care unit.
